# Melioidosis at a Tertiary Care Center in Jaipur, India: Diagnostic Challenges and Therapeutic Insights

**DOI:** 10.7759/cureus.90261

**Published:** 2025-08-16

**Authors:** Sidhya Choudhary, Navya Sharma, Rajni Sharma

**Affiliations:** 1 Microbiology, Sawai Man Singh (SMS) Medical College, Jaipur, IND; 2 Medicine and Surgery, Sawai Man Singh (SMS) Medical College, Jaipur, IND

**Keywords:** antimicrobial therapy, burkholderia pseudomallei, liver abscess, melioidosis, non-endemic region

## Abstract

Melioidosis, caused by *Burkholderia pseudomallei*, is a potentially fatal infectious disease endemic to Southeast Asia and Northern Australia. We report a case of melioidosis in a male farmer in his early 50s from central India, highlighting diagnostic challenges and therapeutic outcomes in a non-endemic region. The patient presented with multiple fever episodes and acute abdominal pain primarily localized to the right upper quadrant. Physical examination revealed hepatomegaly and tenderness in the upper right quadrant. Imaging studies confirmed the presence of multiple liver abscesses. Laboratory investigations revealed leukocytosis and elevated liver enzyme and inflammatory marker levels. *B. pseudomallei* was detected in blood cultures and confirmed by matrix-assisted laser desorption ionization-time of flight (MALDI-TOF) assay. The patient was treated with meropenem and cotrimoxazole, which resulted in a good clinical response.

## Introduction

*Burkholderia pseudomallei* (*B. pseudomallei*), a gram-negative bacterium present in soil, is responsible for melioidosis. This disease is endemic to Southeast Asia and Northern Australia and is becoming more prevalent in other tropical regions with high rainfall [1]. India, the largest nation in South Asia, possesses a favorable environment and a huge diabetic population, making it a potential hotspot for this disease. Melioidosis can manifest as pneumonia, septic arthritis, osteomyelitis, visceral abscesses, and granulomatous lesions, impacting nearly all organ systems. Fulminant sepsis is prevalent and frequently associated with high mortality rates [2].

Melioidosis exhibits a high fatality rate of 19-36% in endemic areas, with worldwide mortality rates ranging from 9% to 70% [2]. This infection impacts both humans and animals and is designated as a category B bioterrorism agent and a tier 1 select agent by the U.S. Centers for Disease Control and Prevention (CDC) [3]. Reports of melioidosis are increasing globally, with an estimated 165,000 cases and 89,000 deaths annually [4].

Presently, there exists limited environmental data regarding the geographic distribution and prevalence of *B. pseudomallei*. This report presents the first documented case from Jaipur in northwestern India.

## Case presentation

A male farmer in his early 50s from central India presented to a tertiary care hospital in northwestern India in October 2023 with a history of acute abdominal pain associated with multiple fever episodes since past two weeks. The pain, primarily localized to the right upper quadrant (RUQ), which occasionally radiates to the right shoulder or back, is described as dull and aching. He also complained of fever, chills, malaise, loss of appetite, and weakness over the past 20 days. There were no changes in the bowel movements or urinary symptoms. The patient had a notable history of chronic alcohol use and binge drinking over a period of 20 years. He occasionally mentioned swimming in the water body right in front of his house. 

Physical examination revealed tenderness in the right upper quadrant, hepatomegaly, and occasionally, a palpable liver edge. There were signs of peritonitis indicating that the abscess had ruptured and referred pain to the right shoulder due to diaphragmatic irritation. Laboratory investigations showed leukocytosis, elevated liver enzymes (aspartate aminotransferase (AST), alanine aminotransferase (ALT), alkaline phosphatase (ALP)), and elevated inflammatory markers (C-reactive protein (CRP), erythrocyte sedimentation rate (ESR)) (Table 1). Aerobic blood culture was negative. Due to persistent fever, elevated leukocyte count, and increased procalcitonin levels, the antibiotic regimen was escalated to intravenous piperacillin-tazobactam (13.5 g daily; 12 g piperacillin/1.5 g tazobactam) for seven days.

**Table 1:** Laboratory parameters.

Parameters	Patient values	Reference range	Units
Complete blood count			
Hemoglobin	8.8	13.00-17.00	g/dL
Red blood cell count	3.22	4.50-5.50	mill/mm^3^
Total leukocyte count	18.72	4.00-10.00	thousand/mm^3^
Neutrophils	15.15	2.00-7.00	thousand/mm^3^
Lymphocytes	1.11	1.00-3.00	thousand/mm^3^
Monocytes	0.26	0.02-1.00	thousand/mm^3^
Eosinophils	0.16	0.02-0.50	thousand/mm^3^
Basophils	0.04	0.02-0.10	thousand/mm^3^
Platelet count	6.32	6.5-12.0	thousand/mm^3^
Liver and kidney panel			
Creatinine	1.33	0.67-1.17	mg/dL
Urea	39.0	14.9-38.5	mg/dL
Aspartate aminotransferase (AST)	115	<50	U/L
Alanine aminotransferase (ALT)	65	<50	U/L
Gamma-glutamyl transpeptidase (GGTP) ​​​​​​​	189	<55	U/L
Bilirubin total	2.30	0.20-1.10	mg/dL
Bilirubin direct	1.21	<0.20	mg/dL
Bilirubin indirect	1.09	<1.10	mg/dL
Total protein	5.60	6.40-8.10	g/dL
Albumin	2.65	3.20-4.60	g/dL
Globulin	2.95	2.0-3.5	gm/dL
Albumin: globulin ratio	0.89	0.90-2.00	-
Inflammatory markers			
Erythrocyte sedimentation rate (ESR)	55	0-20	mm/hour
C-reactive protein (CRP)	74	<6	mg/dL
Procalcitonin	1.02	<0.05	ng/mL

**Figure 1:**
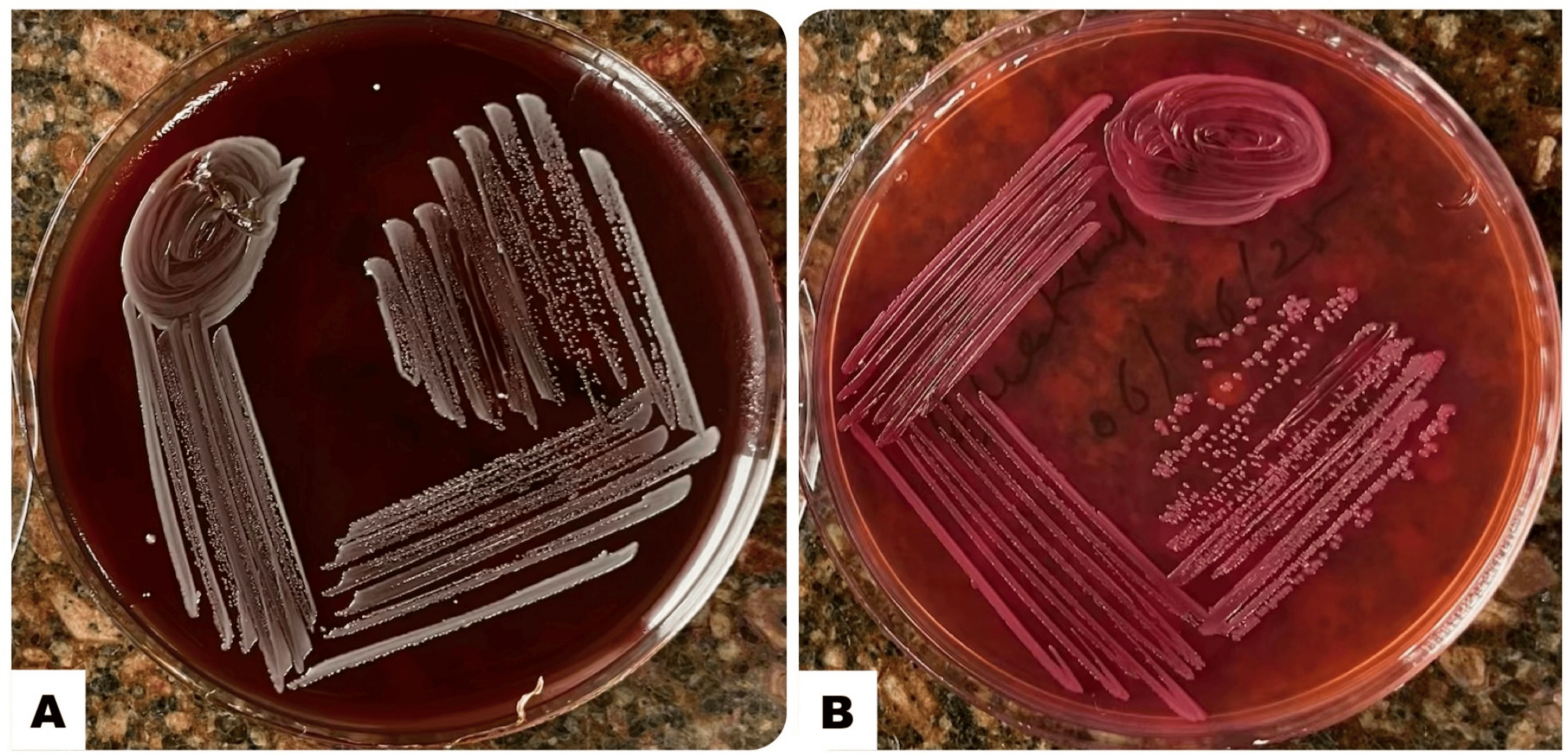
Growth of Burkholderia pseudomallei on culture media. (A) Blood agar showing dry, wrinkled, non-hemolytic colonies with metallic sheen after 48 hours of incubation. (B) MacConkey agar showing dry colonies with pinkish hue, possibly due to delayed lactose oxidation or pigment production, though the organism is typically a non-lactose fermenter.

Ultrasound imaging revealed multiple hypoechoic lesions in the liver, with the largest lesion measuring 65 mm × 47 mm × 53 mm. Ultrasound-guided aspiration yielded 57 cc from the right lobe and 26 cc from the left lobe, confirming the presence of liver abscesses. Histopathological examination ruled out malignancy, with Gram staining of the aspirated pus cells suggestive of an infectious etiology. Routine laboratory testing using Gram stain microscopy and aerobic bacterial culture revealed findings indicative of *Burkholderia *spp., including a metallic sheen observed on blood agar (Figure 1), positive oxidase test results, and a resistance profile showing resistance to gentamicin and colistin, but sensitivity to amoxicillin-clavulanate.

Although the growth is often confused with *Pseudomonas *spp., the matrix-assisted laser desorption ionization-time of flight (MALDI-TOF) assay confirmed *Burkholderia pseudomallei*. Antimicrobial susceptibility testing using the disk diffusion method revealed susceptibility to minocycline, ciprofloxacin, and levofloxacin, with dose-dependent susceptibility to meropenem (Table 2). The isolate showed intrinsic resistance to gentamicin and colistin, consistent with known *Burkholderia pseudomallei* resistance patterns.

**Table 2:** Pyogenic culture and susceptibility. S or sensitive: Indicates the antimicrobial agent is clinically effective when used in standard therapeutic dose. I or intermediate: Indicates the antimicrobial agent may still be clinically effective when used with increased dose/frequency if the patient's vital parameters permit. However, established clinical trials are not available. It may also indicate therapeutic efficacy in physiologically concentrated sites. R or resistant: Indicates clinically ineffective when used in standard or increased therapeutic dose.

S. no	Antibiotics	Zone (mm)	Interpretation
1	Piperacillin/tazobactam	6	Resistant
2	Ceftazidime	6	Resistant
3	Cefepime	6	Resistant
4	Meropenem	18	Intermediate
5	Aztreonam	6	Resistant
6	Colistin	6	Resistant
7	Gentamycin	6	Resistant
8	Minocycline	25	Sensitive
9	Chloramphenicol	6	Resistant
10	Ciprofloxacin	28	Sensitive
11	Levofloxacin	22	Sensitive
12	Cotrimoxazole	6	Resistant

He received intravenous meropenem (1 g IV every eight hours) for four weeks and was discharged in stable clinical condition after completing the IV antibiotic course. At discharge, he was instructed to follow a three-month oral cotrimoxazole regimen (160/800 mg tablets BD) to eradicate *Burkholderia pseudomallei*, with follow-up scheduled after two weeks.

Symptomatic and radiological improvements were observed two weeks after starting treatment. The patient was followed up clinically and radiologically for six weeks until symptom resolution. Since then, the patient has been followed up monthly and has not reported any clinical or radiological evidence of the disease. Complete clinical improvement and near-complete radiological resolution were observed. 

## Discussion

Melioidosis, initially named Whitmore disease, was first reported by Whitmore and Krishnaswami in Rangoon, Burma, in 1912, resembling glanders [5]. It is caused by *Burkholderia pseudomallei* and is predominantly found in endemic regions, such as Singapore, Malaysia, Thailand, and Northern Australia. Infection typically occurs through puncture wounds, ingestion, or inhalation of aerosolized bacteria, with an increased incidence during the rainy season or tropical storms [1,6-8].

A recent study using computer models estimated that approximately 165,000 people worldwide could contract melioidosis each year, with South Asia having the most cases (44% of all cases) [9]. In India, most cases have been reported along the coastal regions in southern states, such as Kerala, Karnataka, and Tamil Nadu, with occasional cases in the eastern and northeastern regions [10-12].

As the largest country in South Asia, India, with its favorable environment and large diabetic population, may be a potential 'hotspot' for melioidosis. However, the actual burden of melioidosis in India remains unknown, owing to limited laboratory resources and awareness. Clinical diagnosis is particularly challenging because the disease can present in various ways and often mimics other infections, such as tuberculosis, which is highly prevalent in developing countries, such as India. 

The risk factors for melioidosis include diabetes mellitus, excessive alcohol consumption (specifically binge drinking), lung pathology, and chronic kidney disease. People in endemic areas with frequent exposure to soil and water are particularly susceptible to infection [13]. The disease can result in severe pneumonia, septicemia, and meningitis, with diabetes and alcohol abuse being identified as significant risk factors [14]. 

Approximately 39% of individuals infected with* Burkholderia pseudomallei* have reported recent episodes of binge drinking, although the exact effect of alcohol on the interaction between *Burkholderia* and the host immune system remains uncertain [9,15]. Both cellular and humoral immunities are critical for defense against this intracellular pathogen, with CD8+ and CD4+ T cells playing vital roles. A study showed that binge alcohol weakened the immune response in mice infected with *B. vietnamiensis* and *B. thailandensis* leading to prolonged infections and higher mortality rates [16]. Conditions related to binge alcohol appear to enhance biofilm production and bacterial persistence, suggesting that alcohol can exacerbate infections by *Burkholderia* spp. Future research will focus on the detailed mechanisms of this immune dysfunction and the potential of alcohol to increase susceptibility to *B. pseudomallei* infections.

This is the first case at our institution illustrating the diagnostic challenges and therapeutic considerations in managing melioidosis, particularly in non-endemic regions. Bedside ultrasound facilitates rapid detection of liver and spleen abscesses, facilitating timely diagnosis [17]. This underscores the importance of prompt recognition and appropriate antimicrobial therapy to achieve favorable outcomes. Further research is warranted to elucidate the epidemiology and clinical profile of melioidosis in India.

The limitations of this case report include its focus on a single patient from a tertiary care center in northwestern India, limiting the generalizability to broader populations within the region. Diagnostic decisions rely on local clinical and laboratory resources, which may differ from settings with varying diagnostic capabilities or resource constraints. The treatment approach and outcomes were based on institutional guidelines and patient responses, potentially influencing outcomes compared to settings with different treatment protocols or antimicrobial resistance patterns. Finally, the follow-up duration of six weeks post-treatment may not capture longer-term outcomes or relapses that could occur with melioidosis management.

## Conclusions

This case highlights the emergence of melioidosis in northwestern India and the challenges of diagnosing and treating it in a non-endemic setting. The detection of *Burkholderia pseudomallei* in a previously unreported region calls for greater clinical awareness and better diagnostic support. Early recognition and prompt antimicrobial therapy are crucial for favorable outcomes. Broader epidemiological research is needed to guide early diagnosis and effective management across India.
